# Brain Stimulation for Psychiatric Disorders: Emerging Evidence and New Perspectives

**DOI:** 10.3390/brainsci15020155

**Published:** 2025-02-04

**Authors:** Jacopo Lisoni, Stefano Barlati

**Affiliations:** 1 Department of Mental Health and Addiction Services, ASST Spedali Civili of Brescia, 25123 Brescia, Italy; stefano.barlati@unibs.it; 2 Department of Clinical and Experimental Sciences, University of Brescia, 25123 Brescia, Italy

Currently, several Non-Invasive Brain Stimulation (NIBS) techniques are available for clinical application in psychiatric disorders (including major depressive disorder, MDD, and obsessive–compulsive disorder) or are under investigation (including schizophrenia, eating, substance use, and neurodevelopmental disorders) [1]. NIBS includes a plethora of different modalities such as Transcranial Magnetic Stimulation, namely repetitive Transcranial Magnetic Stimulation (rTMS) and Theta Burst Stimulation (TBS); transcranial Electrical Stimulation (tES), namely transcranial Direct Current Stimulation (tDCS), transcranial Alternating Current Stimulation (tACS), and transcranial Random Noise Stimulation (tRNS); and non-invasive seizure therapies such as Magnetic Seizure Therapy (MST) and Electro-Convulsive Therapy (ECT). Additionally, transcutaneous auricular Vagus Nerve Stimulation (taVNS) and low-intensity transcranial focused ultrasound (tFUS) emerged as promising new NIBS modalities for the treatment of psychiatric disorders. On the other hand, invasive brain stimulation techniques include Deep Brain Stimulation (DBS) and Vagus Nerve Stimulation (VNS).

While these interventions seem to improve clinical features including cognition and behavioral manifestations, for most psychiatric disorders, the scientific evidence is often heterogeneous [1,2,3]. Thus, further demonstrations of efficacy are needed to produce firm guidelines. With the aim of increasing the current evidence, this Special Issue explored the clinical applications of such NIBS interventions for different psychiatric conditions, especially focusing on disorders such as MDD and schizophrenia. The papers collected in this Special Issue exhibit different levels of evidence, including critical reviews, systematic reviews, open-label studies, and randomized-controlled trials.

The first part of this Editorial focuses on the studies evaluating NIBS effects in schizophrenia. Despite the growing evidence on NIBS efficacy, to date, little is known about the possibility of combining and integrating these experimental interventions with other therapeutic approaches of proven efficacy to foster clinical and functional outcomes in patients with schizophrenia. Bridging this gap, Lisoni and colleagues collected current evidence on the combination between NIBS and evidence-based psychosocial interventions (EBPI), as the latter represents effective non-pharmacological strategies to improve the core symptoms (such as positive, negative, and cognitive symptoms) of this debilitant disorder. In this critical review, it was found that the field of combining NIBS and EBPI was in its very infancy, as only 11 studies were retrieved. While most studies combined tDCS with cognitive activation training with promising results on the improvements of the working memory domain, only a minority of studies combined rTMS or intermittent TBS (iTBS) with more structured EBPI, such as cognitive remediation or family intervention. In this case, divergent and inconclusive results were found. The authors concluded that further efforts are needed to confirm whether NIBS and EBPSI could be effectively integrated as multimodal interventions to improve treatment outcomes and achieve recovery in schizophrenia. Additionally, the authors discussed some methodological issues to be considered to design further dedicated trials.

Among the studies included by Lisoni and colleagues, this Special Issue published preliminary results from the single-blind study by Vergallito and co-workers. Here, iTBS was applied in combination with individualized training on cognitive abilities (based on elements of cognitive remediation) in people with schizophrenia. In detail, iTBS to the left Dorsolateral Prefrontal Cortex (DLPC) was performed as a primer, which is a sequential application where this NIBS modality was applied before the cognitive training to produce addictive effects. As 21 participants were randomized into four groups, the primary aims were to assess the impact of iTBS and cognitive training combination to improve neurocognitive abilities and negative symptoms, comparing the combined approach with iTBS only, cognitive training only, or sham iTBS. Over 3 weeks, participants received 15 daily sessions of active or sham iTBS (20 trains, 600 pulses per session at 100% of active Motor Threshold, MT) followed or not by the individualized cognitive training. Despite the solid methodological background of this study, the authors found preliminary negative results: as stand-alone interventions, iTBS and cognitive training effectively improved negative symptoms and verbal learning and vigilance, respectively; however, the combined intervention (iTBS + cognitive training) did not provide additional benefits, failing to boost clinical improvements. Nevertheless, these results should be interpreted with caution as they are only preliminary.

The second part of this Editorial is focused on the studies that evaluated NIBS effects in MDD, providing in-depth results on protocol optimization, maintenance strategies, and alternative NIBS strategies to the common rTMS/tES. Treating MDD, a standard rTMS course involves stimulation sessions that are generally administered daily, 5 days per week, for 20–30 sessions over 4–6 weeks. However, the scientific debate has recently focused on the possibility of planning stimulation trials of short duration, namely the accelerated repetitive TMS protocols (arTMS) that involve multiple daily sessions over consecutive days. Given the assumption that repeated stimulation within a condensed period and tightly scheduled sessions can yield sustained efficacy, arTMS is aimed at enhancing clinical effects and shortening response times [4,5]. In this scenario, Prodi and colleagues evaluated a sample of Treatment-Resistant Depression patients treated with a standard rTMS protocol (n = 9) or with an arTMS protocol (n = 19). During the 4-week standard protocol, patients received one daily session on the left DLPFC (10 Hz, at 120% of the rMT (resting Motor Thresold, rMT), 3000 stimuli per session). During the arTMS protocol, the patients received two daily sessions for two weeks on the left DLPFC (10 Hz, at the 120% of rMT, 3000 stimuli per session). Assessments were taken at four timepoints: at baseline, 1 week after the end of treatment, 1 month after the end of treatment, and 3 months after the end of treatment. The authors found significant improvement in depressive, anxious, and cognitive symptoms in patients treated with rTMS. Moreover, considering the two rTMS protocols, it was found that the efficacy seemed comparable at 1 week, but a better clinical response was observed at the 3-month follow-up for patients treated with the arTMS protocol: in fact, considering the reduction at the Montgomery Asberg Depression Rating Scale, a greater treatment response was observed in the arTMS group. Taking the results together, the authors observed that, while the improvement in depressive symptoms with the standard protocol was observed after 4 weeks of treatment, the arTMS protocol allowed us to achieve an equally effective response, but in a shorter and quicker time. Thus, these results suggested that reducing the number of treatment days could lead to improved cost-effectiveness, increasing the accessibility of NIBS treatments for depressed patients.

Speculating on the role of maintenance rTMS after an acute course for patients with MDD [6], Turnier-Shea and colleagues performed a naturalistic, open-label observational study to evaluate the effects of once-weekly (OW) or once-fortnightly (OF) rTMS continuation for patients experiencing relapse from MDD. As both high-frequency rTMS to the left DLPFC and low-frequency rTMS over the right DLPFC were performed, the OW rTMS group consisted of 10 patients and the OF rTMS group consisted of 4 patients. Considering the entire study sample, a significant reduction in clinical severity and depressive symptoms scores was found, demonstrating the effectiveness of continuing the rTMS regimen. Moreover, among the patients treated with OW rTMS, significant improvements in clinical severity and depressive symptoms occurred, especially in those patients who were in partial remission or a relapsed condition at the study entry. On the other hand, for the four patients allocated to the OF-rTMS group, clinical improvements were observed but without statistical significance. Taking together the results, the authors concluded that OW rTMS was an effective maintenance regimen in preventing relapses from new depressive episodes, while for the OF rTMS regimen, further investigations are needed to confirm its effectiveness as a maintenance treatment.

Considering innovative NIBS modalities for the treatment of MDD [7], Guo and co-workers evaluated the effects of a 4-week course of transcutaneous auricular Vagus Nerve Stimulation (taVNS) in reducing the severity of depressive and anxious symptoms in patients with MDD through the modulation of the topological organization of the brain. To test this hypothesis, 19 patients underwent resting-state functional MRI before and after the stimulation, and the graph theory method and network-based statistics (NBS) analyses were performed before and after the treatment. After 4 weeks of taVNS, significant improvements in both depressive and anxious symptoms were reported. Moreover, the authors found that patients with MDD had increased global efficiency and decreased characteristic path length (Lp) suggesting a stronger information integration and faster information communication as changes in Lp were related to the improvement in the severity of depressive symptoms. Additionally, patients with MDD exhibited increased Nodal Efficiency (NE) (NE describes the ability of information to transmit from one node to other nodes, with higher values meaning more efficient information transfer between network nodes) and increased Degree Centrality (DC) (DC quantifies the functional connectivity of a node, with higher values representing a stronger influence on other nodes and a greater capacity to communicate information in the network) in the left angular gyrus, a key node of the default mode network (DMN). The NBS findings showed that depressed patients exhibited reduced functional connectivity between the DMN and the frontoparietal network (FPN), between the DMN and the cingulo-opercular network (CON), and between the FPN and the CON. Furthermore, changes in Lp and DC were correlated with changes in Hamilton Depression Rating Scale scores. These results demonstrated that taVNS can effectively improve depressive symptoms by normalizing the disrupted topological network organization in patients with MDD. Furthermore, these data provided new insights into the neural mechanism underlying taVNS treatment in patients with MDD.

The third part of this editorial is focused on the need to personalize NIBS treatments, also providing insight into new stimulation modalities such as transcranial Focused Ultrasound (tFUS) for the treatment of psychiatric disorders [1,7]. Methodological considerations on alternative ways to identify the stimulation site during NIBS trials were provided by Martin and co-workers that aimed to provide more feasible procedures in clinical practice. Indeed, the traditional methods to identify a stimulation site during NIBS are usually based on the International 10-20 EEG system or structural/functional MRI scans. Going beyond these approaches, Martin and colleagues investigated the use of a novel individualized targeting methodology, involving online rTMS during a cognitive task performance to identify an individual’s optimal stimulation site for the left DLPFC. The study had a within-subject, randomized, sham-controlled, single-blinded experimental design and finally involved 33 healthy subjects (HS). Initially, HS received active or sham TMS at 20 Hz at 110% of rMT to five different locations of the left DLPFC. TMS was performed as an online modality, that is, during the performance of the cognitive task (namely, the random letter generation task). The purpose of this first experimental session was to determine an individualized active target site, and an individualized active control site based on participants’ performance on cognitive tasks. Subsequently, the authors tested the efficacy of targeting the individualized sites of the left DLPFC to improve cognitive flexibility performance among HS following a single session of active or sham iTBS (600 pulses, at 110% of rMT). In this latter experiment, iTBS was performed offline, that is, at rest. Unfortunately, the authors found no significant improvement in the cognitive outcomes following a single session of offline iTBS, advising that these negative results did not support the use of this novel individualized targeting methodology to enhance cognitive flexibility.

Additionally, given the idea that the personalization of tDCS parameters could produce better clinical outcomes by reducing inter-individual variability [7], methodological considerations on tDCS dosing are provided by Bhattacharjee and co-workers. In this cross-sectional study, the authors investigated the feasibility of individualizing tDCS doses by simulating the electric field using T1-weighted brain images. Moreover, electric field modeling was used to determine personalized dosages for two conventional and two high-definition (HD) tDCS montages used to target the dorsal and ventral language pathways of the left hemisphere. The sample consisted of 50 patients with dementia, 25 patients with MDD, and 25 HC. These groups were selected because dementia is characterized by significant brain atrophy while MDD typically is not. The authors found that the conventional tDCS montage resulted in more current intensity and less inter-individual variability at the target region of interest (ROI) than the HD configuration. In other words, conventional tDCS montages required less current dosage for personalization. Conversely, targeting the dorsal language pathway, it was found that, in patients with dementia and brain atrophy, HD tDCS configuration required higher current doses, beyond the tolerable range (>4 mA). Summing up, the authors found that HD tDCS montages require a higher personalized dosage in patients with dementia as the current reaching target ROIs was lower than with conventional tDCS. In contrast, no significant differences in current delivery were found between conventional and HD tDCS configurations either in patients with MDD or in HC without atrophy. In conclusion, it was highlighted that, to personalize tDCS parameters, clinicians should consider the level of brain atrophy as stimulation strength in a target ROI could deeply vary according to brain volume loss.

Considering alternative NIBS modalities for the treatment of psychiatric disorders, Keihani and co-workers provided an up-to-date overview of the use of tFUS. In fact, as tFUS can safely and non-invasively stimulate cortical and sub-cortical structures with millimeter precision, this technique offers distinct advantages over other NIBS methods, especially in terms of accessibility to non-cortical regions, and a greater spatial resolution. In this comprehensive narrative review, the authors described the key components of the tFUS system (including tFUS setup and neuronavigational tools used to target deep brain regions) and the protocol parameters to optimize tFUS delivery. Moreover, preliminary experimental findings on tFUS application in psychiatric disorders are provided, especially focusing on MDD, generalized anxiety disorder, substance use disorder, schizophrenia, and autism spectrum disorder. Moreover, MDD represented the most studied psychiatric disorder for tFUS application, highlighting its capability to target subcortical regions, including the cingulate cortex and the thalamus.

Considering other medical conditions usually associated with psychopathological symptoms including mood alterations, Azarkolah and colleagues performed a systematic review of randomized controlled trials with parallel-group design to evaluate tDCS effects in patients with fibromyalgia, especially focusing on pain and fatigue features. As 14 papers were included, the authors found that multiple sessions of tDCS were effective for all included studies, except one in which the improvements were linked to placebo effects. Moreover, the authors provided evidence to suggest tDCS over the primary motor cortex and DLPFC as “effective” and “probably effective”, respectively. In fact, while the modulation of the primary motor cortex would influence sensory pain processing and enhance the descending pain inhibitory system, the stimulation of the DLPFC would lead to the adjustment of the cognitive and emotional aspects of pain due to its connections with the limbic system. Finally, considering the safety profile of this technique in reducing pain perception and fatigue, the authors argued about the feasibility of providing home-based tDCS treatments for patients with fibromyalgia.

This Special Issue additionally gives space to the application of invasive brain stimulation modalities, such as the DBS, since researchers are still trying to identify alternative targeted sites, especially for the treatment of MDD. One of these new targets is the Bed Nucleus of the Stria Terminalis (BNST) as this area seems to have a key role in the regulation of mood and anxiety, given its altered electrophysiological activity observed in patients with MDD. In this context, Fitzgerald and colleagues performed a randomized, double-blind crossover study design, followed by a period of open-label stimulation, to evaluate the efficacy of DBS, with fixed stimulation parameters, targeting the BNST in eight patients with highly refractory MDD. Contrary to previous evidence, the authors found no consistent antidepressant effect of DBS on the BNST across the blinded or open-label follow-up phases. Moreover, significant issues with the tolerability of stimulation parameters were reported. Thus, the authors suggested that other target areas, such as the subgenual anterior cingulate cortex, could represent more fruitful stimulation sites to effectively apply DBS in the case of patients with MDD.

Our Special Issue also provided results from a pre-clinical study to confirm that synaptic plasticity is not only linked to N-methyl-D-aspartate receptor (NMDAR) activation but also involves mechanisms linked to metabotropic glutamate receptor (mGluR) activity. In a study on the rodent model, Holl and colleagues evaluated the interplay between magnetic stimulation and mGluR activation by testing the effect of high-frequency TMS. The Authors confirmed that high-frequency TMS produced synaptic potentiation and that an asynchronous glutamate release during the stimulation could be responsible for mGluR activation.

Concluding, according to the studies included in this Special Issue, we observed a greater scientific interest in the application of NIBS strategies in Psychiatry, especially in conditions such as MDD and schizophrenia. In fact, these severe mental disorders represent the most studied conditions for which NIBS could represent groundbreaking strategies, especially as add-on therapy, to improve the management and the care of patients [3]. Furthermore, by addressing important methodological issues, including protocol optimization and the effects of new NIBS modalities, we hope that this Special Issue could enhance the level of scientific evidence and increase knowledge on NIBS effects, promoting the personalization of NIBS interventions for people living with severe mental disorders.
